# Phase transition and magnetocaloric properties of Mn_50_Ni_42−*x*_Co_*x*_Sn_8_ (0 ≤ *x* ≤ 10) melt-spun ribbons

**DOI:** 10.1107/S2052252517016220

**Published:** 2018-01-01

**Authors:** Zongbin Li, Yiwen Jiang, Zhenzhuang Li, César Fidel Sánchez Valdés, José Luis Sánchez Llamazares, Bo Yang, Yudong Zhang, Claude Esling, Xiang Zhao, Liang Zuo

**Affiliations:** a Key Laboratory for Anisotropy and Texture of Materials (Ministry of Education), School of Materials Science and Engineering, Northeastern University, Shenyang 110819, People’s Republic of China; b División Multidisciplinaria, Ciudad Universitaria, Universidad Autónoma de Ciudad Juárez (UACJ), Calle José de Jesús Macías Delgado No. 18100, Ciudad Juárez, Chihuahua 32579, Mexico; c Instituto Potosino de Investigación Científica y Tecnológica, Camino a la Presa San José 2055, Col. Lomas 4a, San Luis Potosí, S.L.P. 78216, Mexico; d Laboratoire d’Étude des Microstructures et de Mécanique des Matériaux (LEM3), CNRS UMR 7239, Université de Lorraine, Metz 57045, France; e Laboratory of Excellence on Design of Alloy Metals for low-mAss Structures (DAMAS), Université de Lorraine, Metz 57045, France; f Taiyuan University of Science and Technology, Taiyuan, Shanxi 030024, People’s Republic of China

**Keywords:** MnNi-based alloys, melt-spun ribbons, magnetostructural coupling, magnetocaloric effect

## Abstract

The characteristics of magnetostructural coupling play a crucial role in the magnetic field-driven behaviour of magnetofunctional alloys. The availability of magnetostructural coupling over a broad temperature range is of great significance for scientific and technological purposes. This work demonstrates that strong magnetostrucural coupling can be achieved over a wide temperature range (222 to 355 K) in Co-doped high Mn-content Mn_50_Ni_42−*x*_Co_*x*_Sn_8_ (0 ≤ *x* ≤ 10) melt-spun ribbons. It is shown that, over a wide composition range with Co content from 3 to 9 at.%, the paramagnetic austenite first transforms into ferromagnetic austenite at *T*_C_ on cooling, then the ferromagnetic austenite further transforms into a weakly magnetic martensite at *T*_M_. Such strong magnetostructural coupling enables the ribbons to exhibit field-induced inverse martensitic transformation behaviour and a large magnetocaloric effect. Under a field change of 5 T, a maximum magnetic entropy change Δ*S*_M_ of 18.6 J kg^−1^ K^−1^ and an effective refrigerant capacity *RC*_eff_ of up to 178 J kg^−1^ can be achieved, which are comparable with or even superior to those of Ni-rich Ni–Mn-based polycrystalline bulk alloys. The combination of high performance and low cost makes Mn–Ni–Co–Sn ribbons of great interest as potential candidates for magnetic refrigeration.

## Abstract 2

Mn_50_Ni_42−*x*_Co_*x*_Sn_8_ melt-spun ribbons exhibit strong magnetostructural coupling over a wide temperature range from 222 to 355 K. The Δ*S*_M_^peak^ and *RC*_eff_ in Mn_50_Ni_42−*x*_Co_*x*_Sn_8_ melt-spun ribbons are comparable with or even superior to those of Ni-rich Ni–Mn-based polycrystalline bulk alloys.

## 1. Introduction   

The magnetocaloric effect (MCE), characterized in terms of the isothermal magnetic entropy (Δ*S*_M_) or the adiabatic temperature (Δ*T*_ad_) variations, is an intrinsic property of magnetic materials induced by a given value of magnetic field change (μ_0_Δ*H*). Based on this magnetothermal effect, a novel solid-state cooling technology, magnetic refrigeration, is being developed. Compared with conventional gas compression/expansion technology, magnetic refrigeration is environmentally friendly, with zero ozone layer depletion and no global warming contribution. Moreover, it is of higher energy efficiency (over 30%) than is attained by conventional refrigeration (Yu *et al.*, 2003 ▸). For the development of room-temperature active magnetic refrigerators, the development of magnetic materials with large MCE linked to a first-order transition is of great importance. In fact, the search for high-performance magnetocaloric materials in the last 20 years has led to the discovery of several families of materials exhibiting giant MCE, such as Gd–Si–Ge (Pecharsky & Gschneidner, 1997 ▸), La–Fe–Si (Hu *et al.*, 2001 ▸), Fe–Mn–P–As (Tegus *et al.*, 2002 ▸), Fe–Rh (Manekar & Roy, 2008 ▸) and Mn–Ni–(Fe)–Ge (Liu *et al.*, 2012 ▸) alloys, and off-stoichiometric Ni–Mn–*X* Heusler alloys with *X* = Ga, In, Sn or Sb (Krenke *et al.*, 2005 ▸; Planes *et al.*, 2009 ▸; Liu *et al.*, 2012 ▸; Huang *et al.*, 2014 ▸).

Ni–Mn–Sn based Heusler alloys have drawn considerable attention in recent years due to their multifunctional properties that can be controlled by the application of an external magnetic field, such as the magnetic shape-memory effect (MSME) (Kainuma *et al.*, 2006 ▸; Li *et al.*, 2009 ▸), the magnetocaloric effect (Krenke *et al.*, 2005 ▸; Han *et al.*, 2007 ▸; Planes *et al.*, 2009 ▸; Muthu *et al.*, 2010 ▸; Huang *et al.*, 2014 ▸; Ghosh & Mandal, 2014 ▸; Zhang *et al.*, 2015 ▸) and the magnetoresistance (MR) effect (Wang *et al.*, 2008 ▸; Huang *et al.*, 2015 ▸). These remarkable properties are closely related to the martensitic transformation involving coupled structural and magnetization changes, *i.e.* from a ferromagnetic austenite to a weakly magnetic martensite. The transformation is referred as a magneto­structural transformation when the magnetization change Δ*M* is coupled with a crystal structure change. Krenke *et al.* (2005 ▸) first reported a large magnetic entropy change in Ni_50_Mn_50−*x*_Sn_*x*_ alloys with 13 ≤ *x* ≤ 15 (at.%), where the peak Δ*S*_M_ values (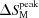) are comparable with those measured for Gd_5_Si_2_Ge_2_ under the same magnetic field change (Pecharsky & Gschneidner, 1997 ▸). Since then, increasing efforts have been made to improve the magnetocaloric properties of these materials through varying the Ni/Mn or Mn/Sn ratio (Krenke *et al.*, 2005 ▸; Han *et al.*, 2007 ▸; Planes *et al.*, 2009 ▸; Muthu *et al.*, 2010 ▸; Ghosh & Mandal, 2014 ▸; Huang *et al.*, 2015 ▸; Zhang *et al.*, 2015 ▸). Some research results suggest that an increase in Mn content contributes significantly to a strong magnetostructural coupling (Krenke *et al.*, 2005 ▸; Han *et al.*, 2007 ▸; Planes *et al.*, 2009 ▸; Muthu *et al.*, 2010 ▸; Ghosh & Mandal, 2014 ▸; Huang *et al.*, 2015 ▸; Zhang *et al.*, 2015 ▸), resulting in enhanced magnetocaloric properties. In this context, high Mn-content Mn–Ni–Sn off-stoichiometric alloys are of interest as magnetocaloric mater­ials.

Based on first-principles calculations, Paul & Ghosh (2011 ▸) predicted the existence of martensitic and magnetic transitions in Mn–Ni–Sn alloys. Ma *et al.* (2012 ▸) experimentally analysed the compositional dependence of the martensitic and magnetic transition temperatures of these ternary alloys. It was then experimentally confirmed that certain alloy compositions may show a strong magnetostructural coupling from weakly magnetic martensite to ferromagnetic austenite (Xuan *et al.*, 2010 ▸; Ma *et al.*, 2012 ▸; Tao *et al.*, 2012 ▸; Ghosh & Mandal, 2013 ▸), which enables them to be potential candidates for magnetic refrigeration applications.

For any magnetic field-induced functional behaviour, the features of the magnetostructural coupling play a key role. To achieve a strong magnetostructural transformation in this type of alloy, the phase transition should occur between a weakly magnetic martensite and a ferromagnetic austenite, which indicates that the Curie temperature (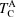) of the austenite should be higher than the martensitic transformation temperature (*T*_M_). However, it is found that the 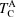 of ternary Mn–Ni–Sn alloys is usually around or below 300 K (Xuan *et al.*, 2010 ▸; Ma *et al.*, 2012 ▸; Tao *et al.*, 2012 ▸; Ghosh & Mandal, 2013 ▸), which strongly limits the working temperature range for room-temperature applications. If the 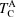 could be enhanced to higher temperatures, the temperature range for the occurrence of the magnetostructural transformation would be enlarged greatly. In facing this challenge, the key issue consists of tuning the martensitic and magnetic transition temperatures while keeping the magnetostructural coupling unchanged over a wide temperature range (Li *et al.*, 2012 ▸; Wei *et al.*, 2015 ▸).

It is worth mentioning that a common way of tuning *T*_M_ and 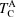 in Ni–Mn based alloys is to introduce a fourth substitutional element. In particular, it has been found that Co doping can not only affect the martensitic and magnetic transformation temperatures but also enhance the ferromagnetic properties of the parent austenitic phase (Ito *et al.*, 2010 ▸; Cong *et al.*, 2010 ▸, 2012 ▸). In addition, Co doping can also bring a great enhancement of the effective magnetic refrigeration capacity *RC*_eff_ through broadening the temperature region of the phase transition (Huang *et al.*, 2014 ▸). In the present investigation, we started from the high Mn-content Mn_50_Ni_42_Sn_8_ alloy and Co was introduced to replace Ni in order to tune the coupled magnetostructural transition. A series of Mn_50_Ni_42−*x*_Co_*x*_Sn_8_ alloys with 0 ≤ *x* ≤ 10 (at.%) were prepared as ribbons by rapid solidification using the melt-spinning technique. Recently, this approach has been successfully applied to the synthesis of Ni–Mn-based alloys (Rama Rao *et al.*, 2007 ▸; Hernando *et al.*, 2008 ▸, 2009 ▸; Sánchez Llamazares *et al.*, 2008 ▸, 2009 ▸; Liu *et al.*, 2009 ▸; Li *et al.*, 2012 ▸, 2014 ▸). The method allows a microstructure refinement and avoids the use of long-term high-temperature post-heat treatments to achieve a highly homogeneous chemical composition. Moreover, it offers an ideal geometric shape for use in refrigeration devices, as the influence of the demagnetizing factor on Δ*S*_M_ could be negligible due to the large aspect ratio when ribbon-shaped refrigerants are magnetized along their longitudinal direction (Caballero-Flores *et al.*, 2009 ▸).

In this work, we demonstrate that a strong magneto­structural coupling can be achieved over a wide temperature range, namely between 222 and 355 K, for Mn_50_Ni_42−*x*_Co_*x*_Sn_8_ melt-spun ribbons. Such strong magnetostructural coupling enables the ribbons to exhibit field-induced inverse martensitic transformation behaviour and a large magnetocaloric effect. Under a field change of 5 T, a maximum magnetic entropy change 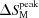 of 18.6 J kg^−1^ K^−1^ and an effective refrigerant capacity *RC*_eff_ of 178 J kg^−1^ were achieved. It is shown that Co doping of Mn–Ni–Sn alloys enables us to obtain a strong magnetostructural coupling over a wide temperature range with enhanced magnetocaloric properties. Thus, Mn–Ni–Co–Sn ribbons are of great interest as potential candidates for magnetic refrigeration.

## 2. Experimental   

Bulk polycrystalline alloys with a nominal composition of Mn_50_Ni_42−*x*_Co_*x*_Sn_8_ (*x* = 0, 1, 2,…, 10 at.%) were prepared by arc-melting under an argon atmosphere using high-purity metal elements (>99.9 wt%). To ensure a good compositional homogenization, the as-cast ingots were flipped over and remelted four times. The ribbons were prepared in a single copper roller melt-spinning equipment from these as-cast precursor ingots. The as-cast alloys were melted by induction heating in a high-purity quartz tube under an argon atmosphere, and then ejected onto the rotating copper wheel at a linear speed of 20 m s^−1^. For microstructural observations, the ribbon plane of the melt-spun ribbons was electrolytically polished with a solution of 20% nitric acid in methanol at ∼273 K. Thin foils for transmission electron microscopy (TEM) observations were electrolytically thinned in a twin-jet device at ∼263 K with the same solution as mentioned above.

The composition of the ribbons was verified by energy-dispersive spectrometry (EDS). The martensitic transformation temperatures were measured by differential scanning calorimetry (DSC) with heating and cooling rates of 10 K min^−1^. The room-temperature crystal structures of the ribbons were identified by X-ray diffraction (XRD) with Cu *K*α radiation and selected-area electron diffraction (SAED). The XRD patterns were measured on the surface of the ribbons. The microstructure characterization was performed using field-emission gun scanning electron microscopy (SEM) (JEOL JSM 7001F) and TEM (JEOL JEM 2100F). The magnetization measurements were carried out using a physical property measurement system (PPMS Dynacool of 9 T, Quantum Design) and a vibrating sample magnetometer (Lakeshore VSM 7407). The magnetic field was applied along the ribbon length direction (rolling direction) to minimize the internal demagnetizing magnetic field. The magnetic entropy change Δ*S*_M_ as a function of temperature was calculated using the Maxwell relation from a set of isothermal magnetization curves *M*(μ_0_ *H*). For the correct determination of the magnetic entropy change across the martensite to austenite transformation, we employed the following thermal protocol to reach each measuring temperature *T*_meas_: under zero magnetic field, the sample was first heated to 400 K to stabilize the austenite, subsequently cooled to 100 K to completely form the martensite, and then heated again to the selected measuring temperature *T*_meas_. For each measurement, the thermal cycle of 400 to 100 K to *T*_meas_ was repeated. Following this procedure, the samples always have the phase constitution corresponding to the thermally induced structural transformation at each *T*_meas_ in the temperature interval of the phase transition (Quintana-Nedelcos *et al.*, 2017 ▸).

## 3. Results   

### 3.1. Crystal structure and microstructure   

EDS measurements were performed to verify the actual compositions of the Mn_50_Ni_42−*x*_Co_*x*_Sn_8_ (0 ≤ *x* ≤ 10) melt-spun ribbons, and the experimentally determined compositions are listed in Table 1 ▸. It is shown that the actual compositions are close to the designed ones.

**Figure 1:**
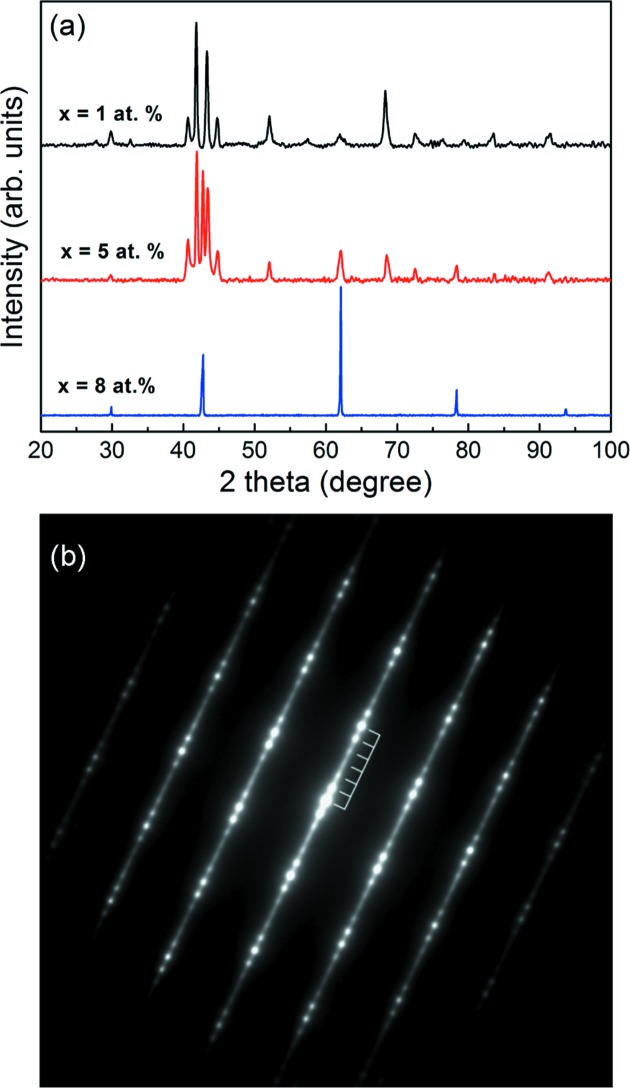
(*a*) Room-temperature XRD patterns for Mn_50_Ni_41_Co_1_Sn_8_ (*x* = 1), Mn_50_Ni_37_Co_5_Sn_8_ (*x* = 5) and Mn_50_Ni_34_Co_8_Sn_8_ (*x* = 8) ribbons. (*b*) Selected-area electron diffraction pattern for 6*M* martensite of Mn_50_Ni_41_Co_1_Sn_8_ ribbons along 〈210〉_M_.

The phase constitutions of the Mn_50_Ni_42−*x*_Co_*x*_Sn_8_ (0 ≤ *x* ≤ 10) melt-spun ribbons were determined from room-temperature XRD patterns. At room temperature, the XRD patterns of the ribbon samples with 0 ≤ *x* ≤ 4 evidence a single martensite state. For *x* = 5 and 6, it is found that the ribbons consist of a mixture of austenite and martensite. On increasing the Co content further, *i.e.* *x* = 7–10, the room-temperature phase of the ribbons turns into a single austenite. Moreover, the martensite diffraction peaks for the ribbons with *x* = 0–6 are located in very close positions, indicating that the addition of Co does not change the crystal structure of the martensite. Typical XRD patterns for the ribbons with *x* = 1, 5 and 8 are shown in Fig. 1 ▸(*a*). Generally, with increasing Co content, the room-temperature phase gradually transforms into the austenite.

It should be noted that, in the 2θ range from 40° to 45°, there are several diffraction peaks in the XRD patterns for the martensite (*e.g.* *x* = 0–6), which may indicate that the crystal structure of the martensite could be a modulated type. In order to further identify the crystal structure of the martensite for the ribbons, SAED measurements were performed. Fig. 1 ▸(*b*) shows a typical SAED pattern along 〈210〉_M_ of the martensite in the Mn_50_Ni_41_Co_1_Sn_8_ ribbon. There are five satellite spots between the two main diffraction spots, indicating that the martensite belongs to a six-layered modulated (6*M*) type with a monoclinic structure (Huang *et al.*, 2015 ▸). Such a 6*M* type crystal structure was also found in an Ni_41_Co_9_Mn_40_Sn_10_ bulk alloy (Huang *et al.*, 2015 ▸). Thus, the martensitic transformation may occur from cubic austenite to monoclinic 6*M* martensite on cooling in the present ribbons.

**Figure 2:**
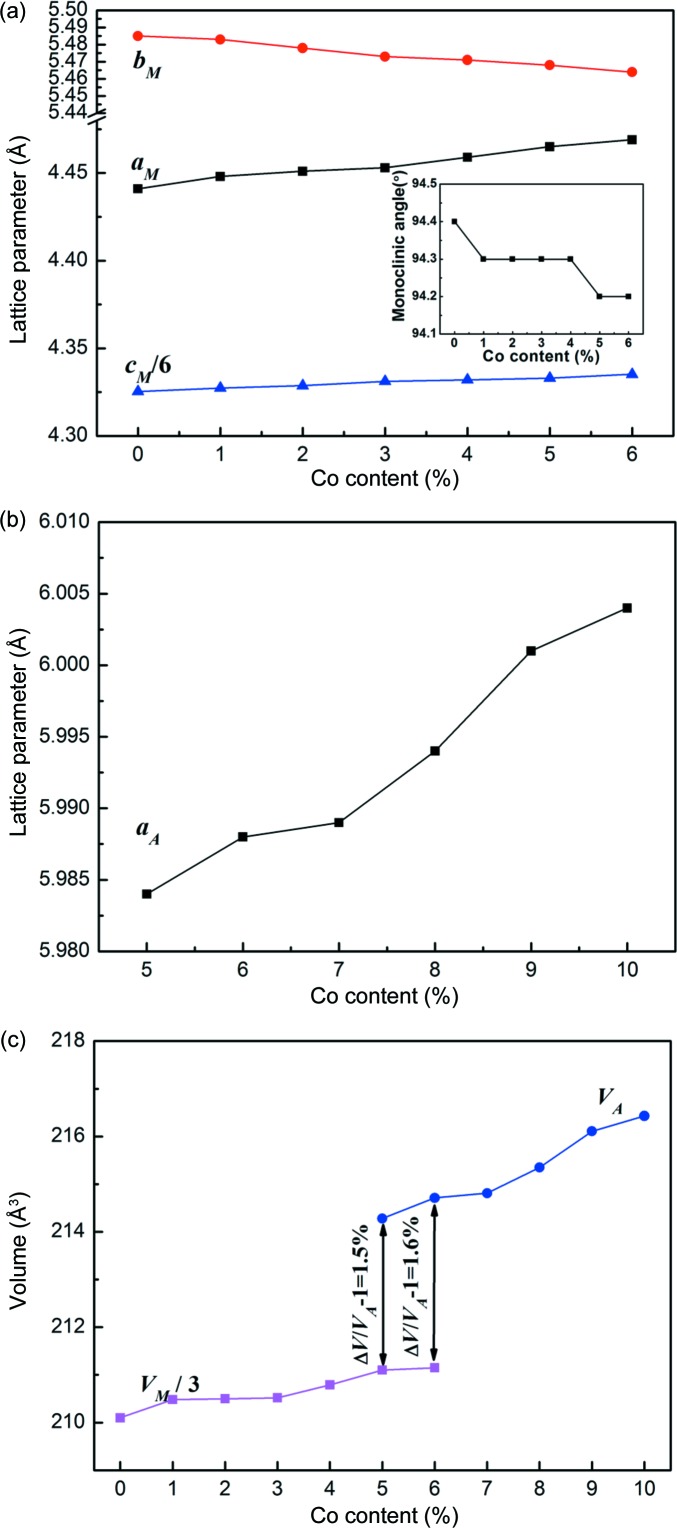
(*a*) The compositional dependence of the lattice parameters for 6*M* martensite for the ribbons with *x* = 0–6. (*b*) The compositional dependence of the lattice parameter for austenite for the ribbons with *x* = 5–10. (*c*) The compositional dependence of the unit-cell volume for austenite and 6*M* martensite.

Fig. 2 ▸(*a*) displays the compositional dependence of the lattice parameters for 6*M* martensite determined from XRD patterns for the ribbons with *x* = 0–6. With increasing Co content, the lattice parameters *a*_M_ and *c*_M_ of the martensite tend to increase monotonically, whereas *b*_M_ decreases. However, the monoclinic angle β seems to be less sensitive to the compositional variation and it is roughly around 94.3°, as shown in the inset of Fig. 2 ▸(*a*). In addition, the lattice parameter *a*_A_ of austenite increases gradually with increasing Co content for the ribbons with *x* = 5–10, as shown in Fig. 2 ▸(*b*). Fig. 2 ▸(*c*) presents the compositional dependence of the unit-cell volume for austenite and 6*M* martensite (*V*_M_/3). Generally, the unit-cell volume increases with increasing Co content for both phases, which is expected due to the larger atomic radius of Co than Ni. Taking into account that the martensitic transformation is a lattice deformation process, certain lattice distortions and unit-cell volume variations are associated with the transformation. Taking the Mn_50_Ni_36_Co_6_Sn_8_ ribbons with coexisting austenite and martensite at room temperature as an example, where the respective lattice parameters of 6*M* martensite and austenite were determined to be *a*_M_ = 4.469 Å, *b*_M_ = 5.464 Å, *c*_M_ = 26.001 Å, β = 94.2° and *a*_A_ = 5.988 Å from the XRD pattern, the martensite lattice contracts by 7.1% along the *b*_M_ axis [*i.e.* (*b*_M_/*a*_A_) − 1] and expands by 5.5% along the *a*_M_ axis [*i.e.* 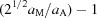] and by 2.4% along the *c*_M_ axis {*i.e.* [(2^1/2^ *c*_M_/6)/*a*_A_] − 1}, considering the lattice correspondence between austenite and 6*M* martensite (Huang *et al.*, 2015 ▸). Such lattice distortion results in a significant unit-cell volume contraction by 1.6% across the martensitic transformation. In addition, it can be expected that the lattice distortion along the *b*_M_ axis would be enlarged with increasing Co content, since *b*_M_ decreases and *a*_A_ increases gradually.

**Figure 3:**
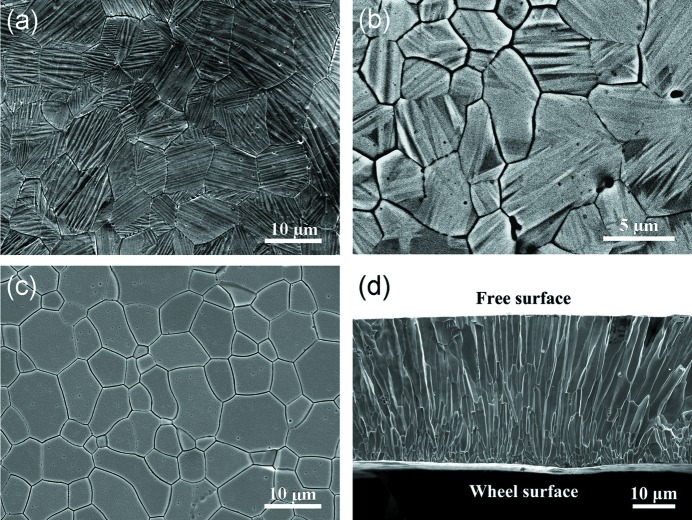
(*a*), (*b*) Backscattered electron (BSE) images of the ribbon plane surfaces for Mn_50_Ni_41_Co_1_Sn_8_ and Mn_50_Ni_37_Co_5_Sn_8_ ribbons, respectively. (*c*), (*d*) Secondary electron (SE) images of the ribbon plane surface for Mn_50_Ni_34_Co_8_Sn_8_ ribbons and of the cross-section for Mn_50_Ni_36_Co_6_Sn_8_ ribbons, respectively.

Figs. 3 ▸(*a*)–3 ▸(*c*) display typical SEM images observed on the ribbon plane surface for Mn_50_Ni_41_Co_1_Sn_8_, Mn_50_Ni_37_Co_5_Sn_8_ and Mn_50_Ni_34_Co_8_Sn_8_ ribbon samples, respectively. For these alloys, the room-temperature phases are a single martensite, a martensite/austenite mixture and a single austenite, respectively. At room temperature, the 6*M* martensite has a plate shape and these martensite plates are clustered in colonies within the austenite grains, whereas the austenite grains have an equiaxial shape in the ribbon plane. Notably, the austenite grain size in the ribbons has been significantly refined due to an ultra-high cooling rate of the melt-spun technique (Quintana-Nedelcos *et al.*, 2013 ▸) compared with the bulk alloys. Fig. 3 ▸(*d*) shows a typical secondary electron (SE) image taken from a cross section perpendicular to the ribbon plane for Mn_50_Ni_37_Co_5_Sn_8_ (*x* = 5) ribbons. The initial austenite grains have a columnar shape, growing approximately perpendicular to the ribbon plane. This morphology is attributed to the effect of the specific heat-transfer conditions of the melt-spun process on grain nucleation and growth (Li *et al.*, 2012 ▸). As expected, at the ribbon surface in contact with the wheel, the average grain size is much smaller than that in the free surface side due to the faster heat extraction, which is consistent with previous observations (Li *et al.*, 2012 ▸).

**Figure 4:**
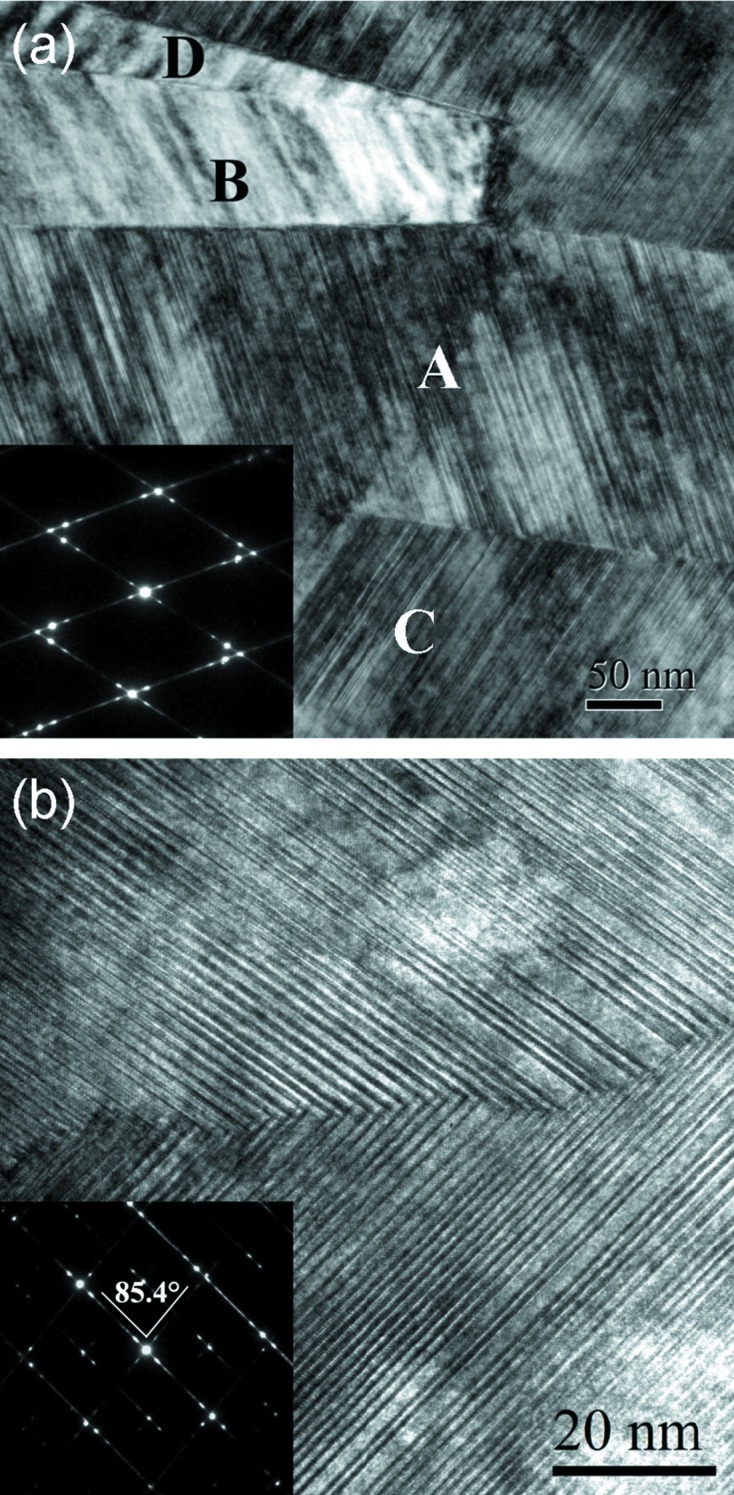
(*a*) A typical bright-field image of 6*M* martensite in one variant colony. The inset shows the SAED pattern along the 〈210〉_M_ direction for variants A and C. (*b*) A typical TEM image for the compound twin interface. The inset shows the corresponding SAED pattern along the 〈010〉_M_ direction for the compound twin.

To analyse further the microstructural features of 6*M* martensite in the ribbons, TEM observations were performed. Fig. 4 ▸(*a*) shows a typical bright-field image of 6*M* martensite taken for Mn_50_Ni_41_Co_1_Sn_8_ ribbons. It is seen that the 6*M* martensite plates exhibit stacking faults as the substructure, which is similar to what is found in other Ni–Mn-based alloys (Sutou *et al.*, 2004 ▸; Nishida *et al.*, 2008 ▸; Li *et al.*, 2016 ▸). There are four types of martensite variants, designated A, B, C and D, distributed alternately within one variant colony. This is similar to the observation for 7*M* martensite in Ni–Mn–Ga alloys (Nishida *et al.*, 2008 ▸; Li *et al.*, 2010 ▸, 2011 ▸). Detailed crystallographic analyses show that adjacent variants could be twin-related to each other, and their twin relationships can be classified into three categories according to the definition of twinning (Christian & Mahajan, 1995 ▸; Nishida *et al.*, 2008 ▸; Zhang *et al.*, 2010 ▸), *i.e.* a type I twin for variant A and variant C (or B and D), a type II twin for A and B (or C and D), and a compound twin for A and D (or B and C), which is consistent with what is found in Ni–Mn–Ga 7*M* martensite (Nishida *et al.*, 2008 ▸; Li *et al.*, 2011 ▸). These crystallographic similarities in variant number and twinning type should be attributed to identical crystal structure types, *i.e.* a monoclinic crystal structure. For the type I twin (A and C or B and D), the twinning plane was determined to be rational {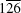}_M_. For the type II twin (A and B or C and D), the twinning direction was determined to be rational 〈
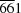
〉_M_. For the compound twin (A and D or B and C), the twinning plane and twinning direction were determined to be {106}_M_ and 〈
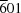
〉_M_, respectively. Moreover, both the type I and type II twin interfaces are straight, whereas the compound twin interface exhibits a stepped structure, as shown in Fig. 4 ▸(*b*). It is noted that the lattice streaks of the basal planes of two variants overlap at the interface. There are many step- and ledge-like structures at the interface. Such crystallographic features are similar to those found in Ni–Mn–Ga modulated martensite (Nishida *et al.*, 2008 ▸). In addition, the monoclinic angle β determined from the SAED pattern along 〈010〉_M_ of the 6*M* martensite (inset of Fig. 4 ▸ *b*) is ∼94.6°, which is consistent with the XRD results and further confirms the monoclinic crystal structure of martensite.

### 3.2. Phase transformation   

**Figure 5:**
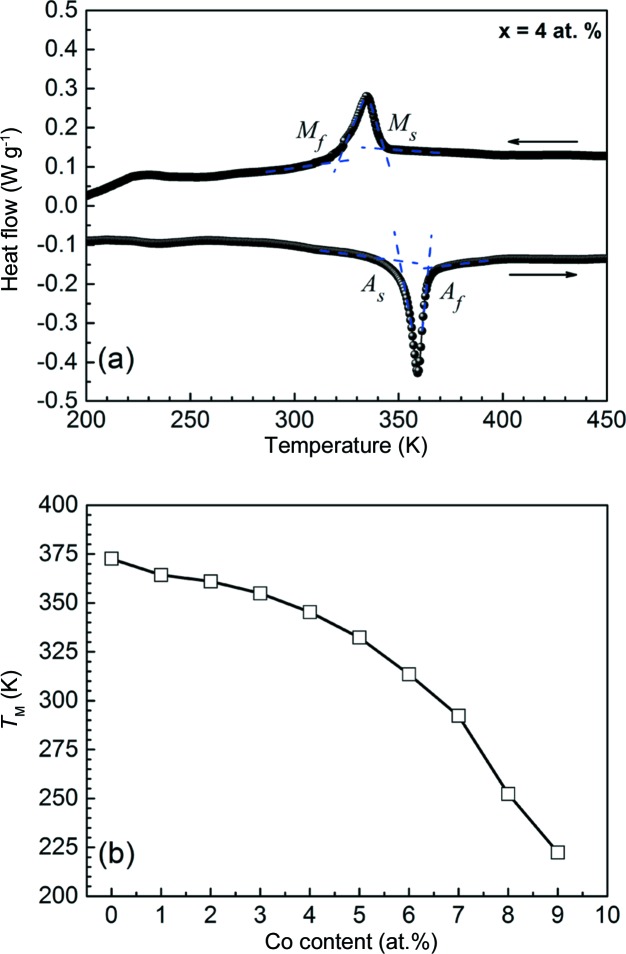
(*a*) DSC curves for Mn_50_Ni_38_Co_4_Sn_8_ ribbon samples. (*b*) The compositional dependence of *T*_M_ for Mn_50_Ni_42−*x*_Co_*x*_Sn_8_ ribbons, with *x* from 0 to 9.

DSC measurements were performed to determine the martensitic transformation temperatures of Mn_50_Ni_42−*x*_Co_*x*_Sn_8_ ribbons. Fig. 5 ▸(*a*) shows representative DSC curves for ribbon samples with *x* = 4, *i.e.* Mn_50_Ni_38_Co_4_Sn_8_. The exothermic and endothermic peaks on the cooling and heating paths denote the occurrence of a reversible martensitic transformation. Through the tangent method, the martensitic transformation start and finish temperatures (*M*_s_ and *M*_f_, respectively) and the inverse transformation start and finish temperatures (*A*_s_ and *A*_f_, respectively) of the ribbons with *x* = 0–9 were well determined from the DSC curves. For the ribbons with *x* = 10, the martensitic transformation temperatures could not be detected down to 150 K (the low-temperature limit of the DSC instrument employed), indicating that the martensitic transformation may occur below this temperature.

Fig. 5 ▸(*b*) displays the compositional dependence of the martensitic transformation temperatures *T*_M_, defined as (*M*_s_ + *M*_f_ + *A*_s_ + *A*_f_)/4, for Mn_50_Ni_42−*x*_Co_*x*_Sn_8_ ribbons with *x* = 0–9. Increasing Co content leads to a gradual decrease in the martensitic transformation temperature, which is consistent with previous results reported for Co-doped Ni–Mn–Sn bulk alloys (Cong *et al.*, 2010 ▸, 2012 ▸). Thus, Co substitution for Ni tends to stabilize austenite in the studied Mn_50_Ni_42−*x*_Co_*x*_Sn_8_ ribbons.

**Figure 6:**
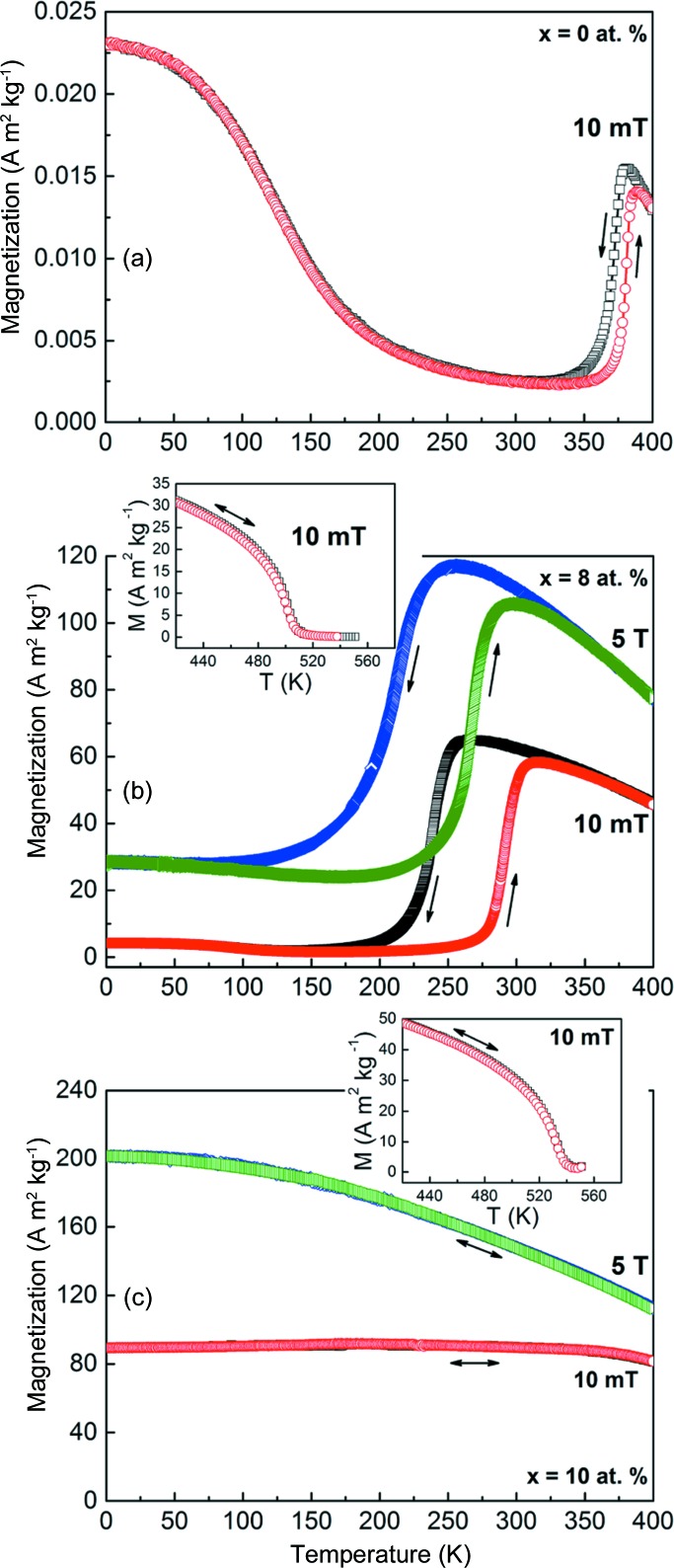
(*a*) *M*(*T*) curves at 10 mT for Mn_50_Ni_42_Sn_8_ ribbons. (*b*) *M*(*T*) curves at 10 mT and 5 T for Mn_50_Ni_34_Co_8_Sn_8_ ribbons. (Inset) *M*(*T*) curves at 10 mT through the ferromagnetic to paramagnetic transition of austenite. (*c)* *M*(*T*) curves for Mn_50_Ni_32_Co_10_Sn_8_ ribbons at 10 mT and 5 T. (Inset) *M*(*T*) curves at 10 mT through the ferromagnetic to paramagnetic transition of austenite.

The magnetization characteristics associated with the phase transition for Mn_50_Ni_42−*x*_Co_*x*_Sn_8_ ribbons were determined from thermomagnetic curves *M*(*T*). The measurements show that a martensitic transformation occurs from paramagnetic austenite to a weakly magnetic martensite for ribbons with *x* = 0, 1 and 2. Typical *M*(*T*) curves for ribbon samples with *x* = 0 (*i.e.* Mn_50_Ni_42_Sn_8_ ribbons) under a field of 10 mT are presented in Fig. 6 ▸(*a*). In this figure, the abrupt magnetization changes on cooling and heating are attributed to the forward and inverse martensitic transformations. The significant thermal hysteresis between the cooling and heating processes confirms the first-order nature of the martensitic transformation. Note that there is no obvious magnetization difference associated with the martensitic transformation.

For the ribbons with *x* from 3 to 9, the *M*(*T*) curves reveal that the paramagnetic to ferromagnetic transition occurs prior to the martensitic transformation. The martensitic transformation then occurs from a ferromagnetic austenite to a weakly magnetic martensite. Typical *M*(*T*) curves for the ribbons with *x* = 8 under fields of 10 mT and 5 T are shown in Fig. 6 ▸(*b*). It can be seen that there is a large magnetization difference [Δ*M* ≃ 92 A m^2^ kg^−1^ as determined from the *M*(*T*) curves under the field of 5 T] associated with the martensitic transformation, suggesting a strong magnetostructural coupling. Compared with the ternary Mn–Ni–Sn alloys, *e.g.* Δ*M* ≃ 60 A m^2^ kg^−1^ in the Mn_50_Ni_40_Sn_10_ alloy (Ma *et al.*, 2012 ▸), the Δ*M* between austenite and martensite in the Mn_50_Ni_34_Co_8_Sn_8_ ribbons has been greatly enhanced by the addition of Co.

It is noted that the martensitic transformation is shifted to a lower temperature region under a field of 5 T, indicating that the inverse martensitic transformation can be induced by a magnetic field. By comparison, *A*_s_ is reduced by ∼27.5 K under a field of 5 T, at a rate of 5.5 K T^−1^. According to the Clausius–Clapeyron relation in the magnetic phase diagram, the decrease in phase transformation temperature induced by the magnetic field can be expressed as Δ*T* ≃ (Δ*M*/Δ*S*)Δ*H*, where Δ*S* and Δ*M* stand for the differences in entropy and magnetization between the austenite and martensite phases, respectively. Apparently, a large Δ*M* and a small Δ*S* will lead to a large Δ*T* and thus benefit the field-induced inverse martensitic transformation behaviour at a constant temperature close to *A*_s_. For the ribbons with *x* = 8, Δ*S* was determined to be 17 J kg^−1^ K^−1^ from DSC measurements. Thus, under a field change of 5 T, the Δ*T* value estimated from the Clausious–Clapeyron equation is 27 K, which is consistent with the experimentally observed temperature shift of ∼27.5 K.

The inset of Fig. 6 ▸(*b*) presents additional *M*(*T*) measurements under a field of 10 mT between 350 and 550 K in order to reveal the paramagnetic to ferromagnetic transition of austenite. Here, the ferromagnetic to paramagnetic transition temperature (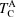) was determined to be ∼501 ± 1 K from the minimum of the d*M*/d*T* *versus* *T* curve, which is obviously higher than that of the ternary Mn–Ni–Sn alloys (Xuan *et al.*, 2010 ▸; Ma *et al.*, 2012 ▸; Tao *et al.*, 2012 ▸; Ghosh & Mandal, 2013 ▸).

Fig. 6 ▸(*c*) presents the *M*(*T*) curves at low and high magnetic field for the ribbon samples with *x* = 10. Notice that austenite remains stable down to 2 K. Thus, the martensitic transformation is totally suppressed over the entire temperature range. The suppression of the martensitic transformation was in fact also observed in Ni–Co–Mn–Sn and Ni–Co–Mn–Ga alloys with high Co content (Cong *et al.*, 2008 ▸; Fabbrici *et al.*, 2009 ▸). The inset of Fig. 6 ▸(*c*) shows the *M*(*T*) curves at 10 mT between 350 and 520 K. It can be seen that the paramagnetic to ferromagnetic transition occurs at 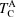 ≃ 533 ± 1 K.

**Figure 7:**
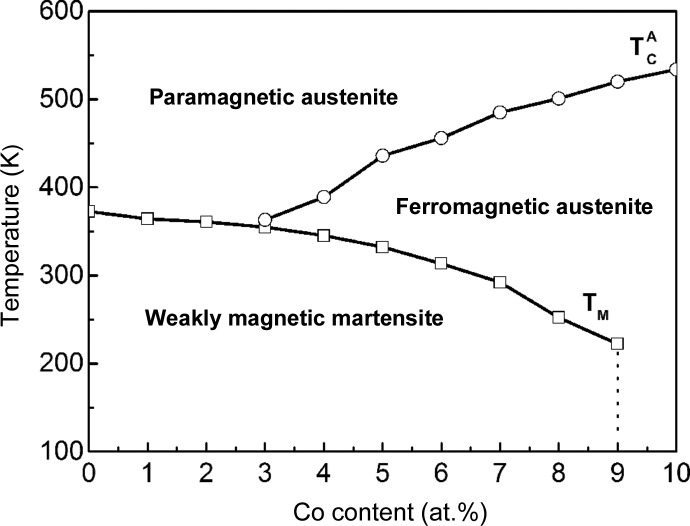
Phase diagram showing the compositional dependence of the martensitic transformation and magnetic transition in Mn_50_Ni_42−*x*_Co_*x*_Sn_8_ melt-spun ribbons with 0 ≤ *x* ≤ 10.

Based on the determined martensitic transformation and magnetic transition temperatures, we constructed a magnetic phase diagram for the series of Mn_50_Ni_42−*x*_Co_*x*_Sn_8_ ribbons (shown in Fig. 7 ▸). With increasing Co content, the martensitic transformation temperature decreases and the magnetic transition temperature increases. According to the magnetic state and phase constitution, the phase diagram can be divided into three regions, paramagnetic austenite, ferromagnetic austenite and weakly magnetic martensite. For the ribbons with *x* from 0 to 2, the martensitic transformation occurs from paramagnetic austenite to weakly magnetic martensite. For the ribbons with *x* from 3 to 9, the paramagnetic austenite first transforms into ferromagnetic austenite at 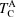 on cooling and then the ferromagnetic austenite transforms into weakly magnetic martensite at *T*_M_. Within this composition range, the ribbons exhibit magnetostructural coupling over a wide temperature range from 355 to 222 K. Moreover, with increasing Co content, the difference between 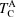 and *T*_M_ becomes larger, widening the ferromagnetic austenite region. For the ribbons with *x* = 10, the paramagnetic austenite transforms on cooling into the ferromagnetic austenite at 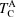. As stated before, the structural transformation could not be detected with the available instrument.

### 3.3. Magnetocaloric properties   

**Figure 8:**
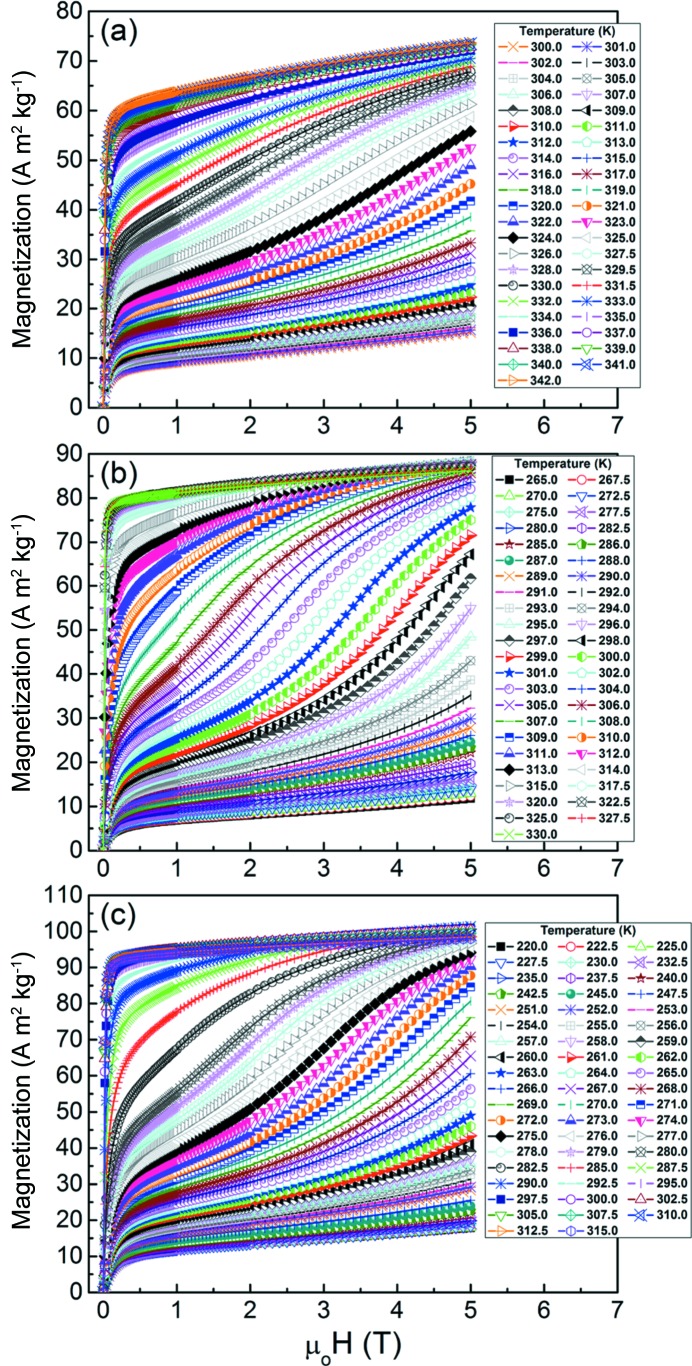
Isothermal magnetization curves across the magnetostructural (martensite to austenite) transformation with a maximum applied magnetic field of μ_0_ *H*_max_ = 5 T for (*a*) Mn_50_Ni_36_Co_6_Sn_8_, (*b*) Mn_50_Ni_35_Co_7_Sn_8_ and (*c*) Mn_50_Ni_34_Co_8_Sn_8_ ribbons.

According to the thermomagnetic measurements, the Mn_50_Ni_42−*x*_Co_*x*_Sn_8_ ribbons with *x* from 6 to 8 possess a relatively high magnetization difference across the martensitic transformation. Thus, further magnetization measurements were performed in order to characterize the magnetic field-induced magnetic entropy change across the inverse martensitic transformation of these alloy ribbons. Figs. 8 ▸(*a*)–8 ▸(*c*) display the measured magnetization isotherms up to a maximum applied magnetic field of μ_0_ *H*_max_ = 5 T. With increasing temperature under a given magnetic field, the magnetization gradually increases across the inverse martensitic transformation, showing the transition from weakly magnetic martensite to ferromagnetic austenite. It is noted that the magnetization isotherms exhibit the characteristics of the *meta*-magnetic transition in the vicinity of the inverse martensitic transformation. At a certain critical field value μ_0_ *H*_cr_, a sudden deviation in the slope is observed, from a linear increase in the magnetization to a faster than linear increase, due to the magnetic field-induced phase transformation from weakly magnetic martensite to ferromagnetic austenite.

**Figure 9:**
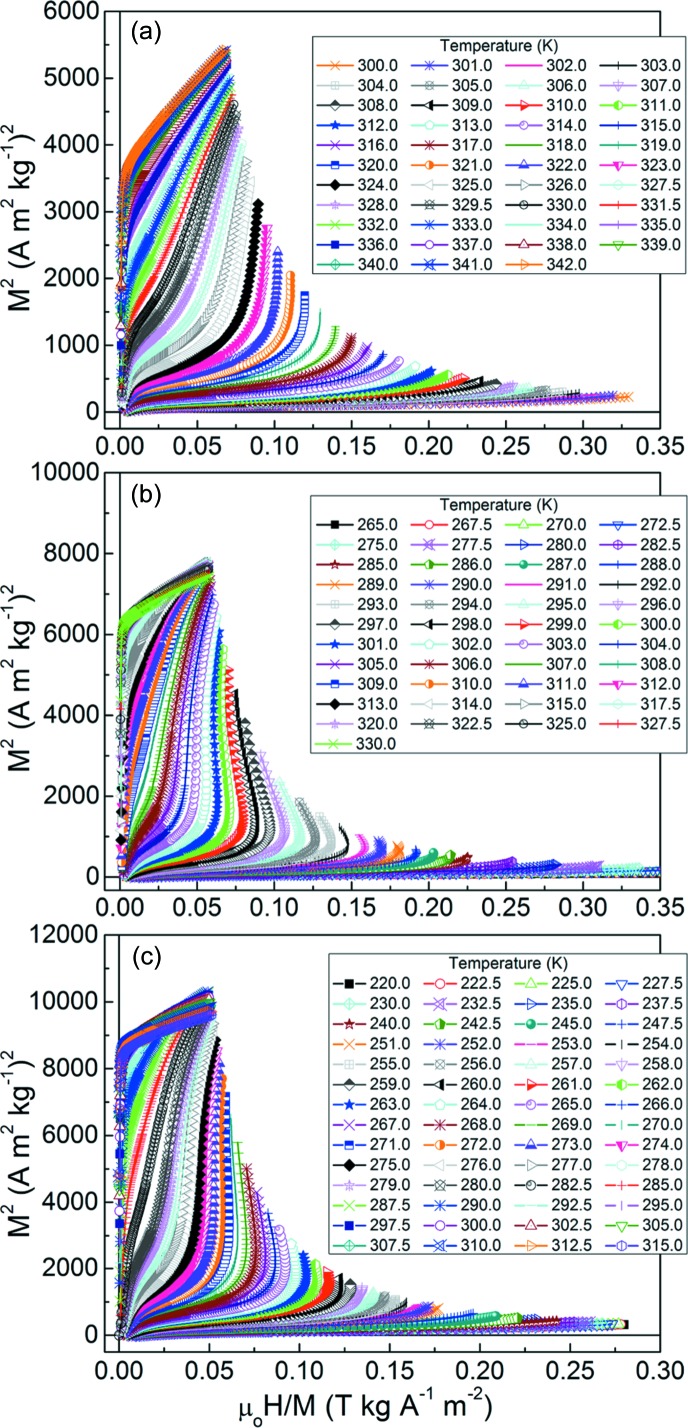
Arrott plots for the melt-spun ribbon samples. (*a*) Mn_50_Ni_36_Co_6_Sn_8_, (*b*) Mn_50_Ni_35_Co_7_Sn_8_ and (*c*) Mn_50_Ni_34_Co_8_Sn_8_.

From the measured sets of isothermal magnetization curves, the corresponding Arrott plots were obtained, as shown in Figs. 9 ▸(*a*)–9 ▸(*c*) for the ribbons with *x* = 6, 7 and 8. The appearance of negative slopes and the trend to draw an S-shape further highlight the first-order nature of the coupled magnetostructural transition.

**Figure 10:**
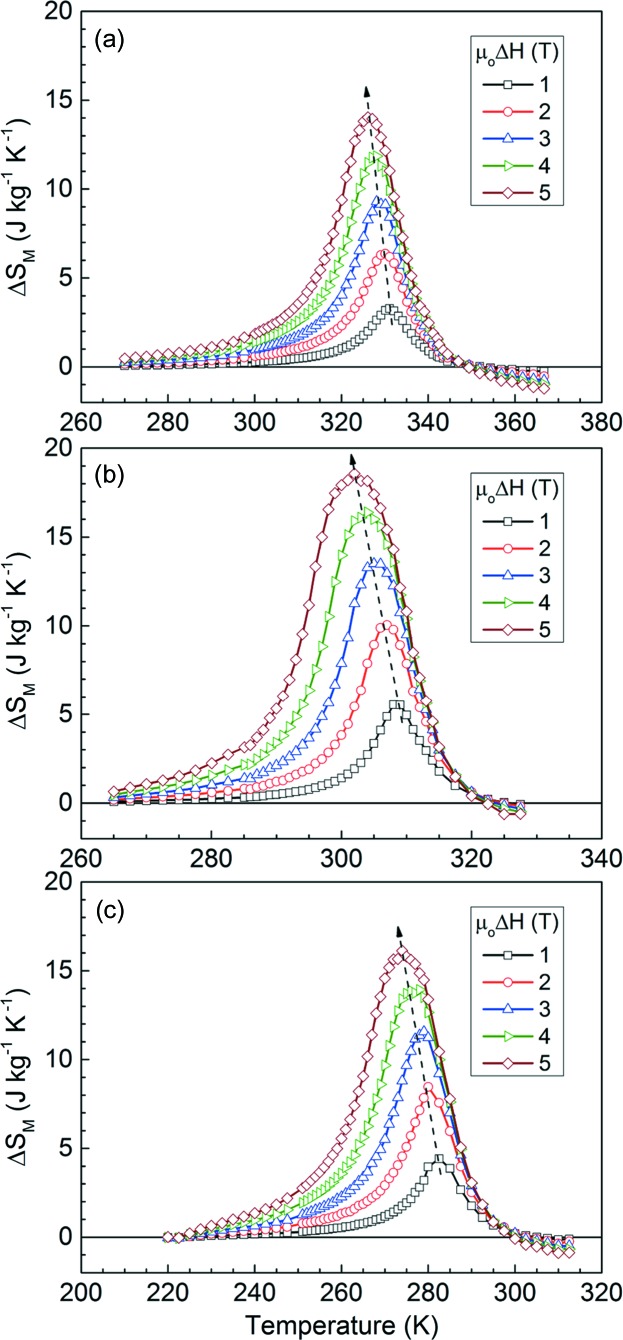
Magnetic entropy change Δ*S*_M_ as a function of temperature through the inverse martensitic transformation for magnetic field changes ranging from 1 to 5 T for (*a*) Mn_50_Ni_36_Co_6_Sn_8_, (*b*) Mn_50_Ni_35_Co_7_Sn_8_ and (*c*) Mn_50_Ni_34_Co_8_Sn_8_ ribbon samples.

Fig. 10 ▸ shows the calculated isothermal magnetic entropy change curves for magnetic fields varying in the range from 1 to 5 T. The ribbons exhibit a large positive Δ*S*_M_ (*i.e.* inverse MCE), in agreement with the large magnetization change from the low-temperature martensite in a weakly magnetic state to the high-temperature ferromagnetic austenite with a strong magnetization. Moreover, in accordance with the field-induced inverse martensitic transformation behaviour, the Δ*S*_M_ peak position shifts to lower temperatures with increasing magnetic field change (as indicated by the dashed lines in Fig. 10 ▸). Under field changes of 2 and 5 T, the 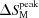 values for the ribbons with *x* = 6, 7 and 8 were determined to be 6.4 (2 T) and 14.0 J kg^−1^ K^−1^ (5 T), 10.0 (2 T) and 18.6 J kg^−1^ K^−1^ (5 T), and 8.5 (2 T) and 16.1 J kg^−1^ K^−1^ (5 T), respectively. For μ_0_Δ*H* = 5 T, the measured values of 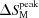 are comparable with those reported for the bulk Ni-rich alloys Ni_50_Mn_37_Sn_13_ (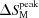 = 15 J kg^−1^ K^−1^; Krenke *et al.*, 2005 ▸), Ni_50_Mn_35_Sn_15_ (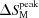 = 18 J kg^−1^ K^−1^; Krenke *et al.*, 2005 ▸) and Ni_40_Co_10_Mn_40_Sn_10_ (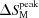 = 14.9 J kg^−1^ K^−1^; Huang *et al.*, 2014 ▸), and for the melt-spun ribbons Ni_48_Mn_39.5_Sn_12.5_ (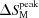 = 15 J kg^−1^ K^−1^; Czaja *et al.*, 2014 ▸) and Ni_43_Mn_42_Co_4_Sn_11_ (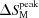 = 19.7 J kg^−1^ K^−1^; Bruno *et al.*, 2014 ▸), and better than those of the bulk Mn-rich alloys Mn_50_Ni_40_Sn_10_ (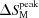 = 8.6 J kg^−1^ K^−1^; Sharma & Suresh, 2015 ▸) and Mn_50_Ni_39_Co_1_Sn_10_ (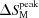 = 10.5 J kg^−1^ K^−1^; Sharma & Suresh, 2015 ▸).

For a better assessment of the magnetocaloric behaviour of the studied ribbons, we calculated the refrigeration capacity *RC*, which represents the amount of thermal energy that can be transferred by the magnetic refrigerant between the cold (*T*_cold_) and hot (*T*_hot_) sinks in one ideal thermo­dynamic cycle. *RC* is defined as 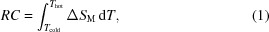
where *T*_hot_ and *T*_cold_ define the full width at half-maximum (δ*T*_FWHM_) of the Δ*S*_M_(*T*) curve. For μ_0_Δ*H* = 2 and 5 T, the determined *RC* values for the ribbon samples with *x* = 6, 7 and 8 were 65 (2 T) and 189 J kg^−1^ (5 T), 86 (2 T) and 259 J kg^−1^ (5 T), and 90 (2 T) and 273 J kg^−1^ (5 T), respectively.

**Figure 11:**
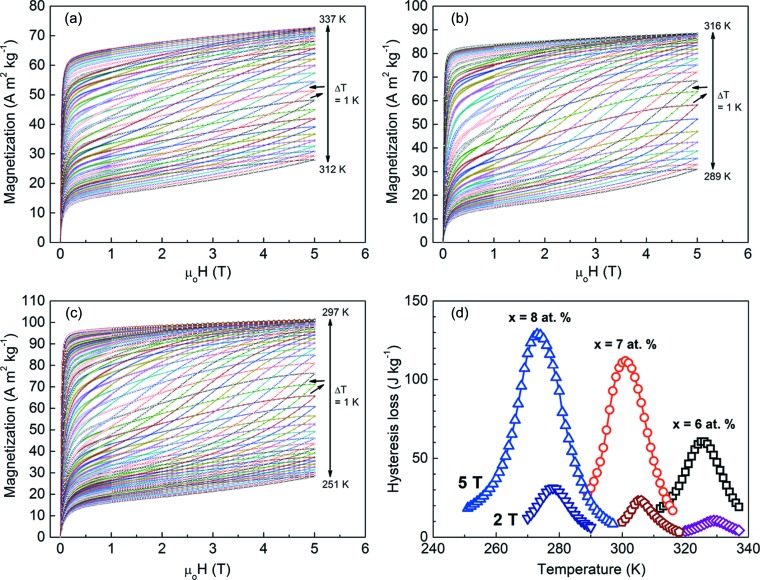
(*a*)–(*c*) Isothermal magnetization curves measured on increasing and decreasing the magnetic field up to μ_0_ *H*_max_ = 5 T through the inverse martensitic transformation for (*a*) Mn_50_Ni_36_Co_6_Sn_8_, (*b*) Mn_50_Ni_35_Co_7_Sn_8_ and (*c*) Mn_50_Ni_34_Co_8_Sn_8_ ribbon samples. (*d*) The temperature dependence of the hysteresis losses for field changes of 2 and 5 T.

In order to determine the effective refrigeration capacity *RC*_eff_, the hysteresis losses were considered. With such a purpose, the field-up and field-down isothermal magnetization *M*(μ_0_ *H*) curves with a maximum field of 2 T (not presented) and 5 T (Figs. 11 ▸ *a* to 11 ▸ *c* for the ribbons with *x* = 6, 7 and 8, respectively) were recorded in the temperature range of the inverse martensitic transformation. The hysteresis loss values were obtained by calculating the areas enclosed between the field-up and field-down *M*(μ_0_ *H*) curves. The hysteresis losses for μ_0_Δ*H* = 2 T and 5 T as a function of temperature are displayed in Fig. 11 ▸(*d*). For the ribbon samples with *x* = 6, 7 and 8, the average losses 〈*HL*〉 were about 8 (μ_0_Δ*H* = 2 T) and 46 J kg^−1^ (μ_0_Δ*H* = 5 T), 17 (2 T) and 84 J kg^−1^ (5 T), and 23 (2 T) and 95 J kg^−1^ (5 T), respectively. *RC*_eff_ values were obtained by subtracting 〈*HL*〉 from *RC*. For the ribbons with *x* = 6, 7 and 8, they were 57, 69 and 67 J kg^−1^, respectively, for μ_0_Δ*H* = 2 T, and 143, 175 and 178 J kg^−1^, respectively, for μ_0_Δ*H* = 5 T. Table 2 ▸ summarizes the magnetocaloric properties under field changes of 2 and 5 T for the studied ribbon samples. The present *RC*_eff_ values for μ_0_Δ*H* = 5 T are comparable with those measured in the bulk Ni-rich alloys Ni_45_Co_5_Mn_36.6_In_13.4_ (198 J kg^−1^; Chen *et al.*, 2012 ▸) and Ni_50_Mn_25_In_25_ (167.5 J kg^−1^; Brock & Khan, 2017 ▸), and higher than those reported for the alloys Ni_50_Mn_33_Cr_1_In_16_ (90 J kg^−1^; Sharma *et al.*, 2011 ▸) and Ni_50_Mn_34_In_16_ (103.8 J kg^−1^; Sharma *et al.*, 2007 ▸). The measured *RC*_eff_ values are also higher than those found in Ni_50_Mn_37_Sn_13_ melt-spun ribbons (54 J kg^−1^; Phan *et al.*, 2012 ▸), Ni_50_Mn_36_Sn_14_ melt-spun ribbons (69 J kg^−1^; Phan *et al.*, 2012 ▸) and Ni_43_Mn_46_Sn_11_ ribbons (115.4 J kg^−1^, Zhang *et al.*, 2015 ▸).

## 4. Discussion   

In general, the valence-electron concentration (*e*/*a*) is a decisive factor for the martensitic transformation temperatures in Ni–Mn-based alloys (Chernenko, 1999 ▸). Increasing the valence-electron concentration can result in increasing the martensitic transformation temperatures and *vice versa*. Here, the number of valence electrons is calculated as the number of 3*d* and 4*s* electrons (Ni 3*d* ^8^4*s* ^2^ and Co 3*d* ^8^4*s* ^1^). The substitution of Ni by Co thus effectively decreases the number of valence electrons. Therefore, in the present Mn_50_Ni_42−*x*_Co_*x*_Sn_8_ series, the martensitic transformation temperature decreases with increasing Co content, which is well in accordance with the general rule that the martensitic transformation temperature decreases with decreasing *e*/*a*. On the other hand, the enhancement of 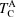 by substitution of Ni with Co could be attributed to the enhanced magnetic exchange interaction (Ma *et al.*, 2008 ▸). The doped Co atoms tend to increase the Mn—Mn distance and thus tune the antiferromagnetically exchange-coupled Mn–Mn moments into ferromagnetically exchange-coupled ones. Accordingly, the magnetization of austenite is significantly increased with increasing Co content, resulting in enhanced Δ*M* accompanying the martensitic transformation and thus strong magnetostructural coupling.

Ni–Mn-based alloys exhibit a significant magnetic entropy change in the vicinity of the magnetostructural transformation. From the view point of practical application, it is of great importance that the working substance can operate over a wide temperature region. Through the substitution of Ni by Co in the studied Mn_50_Ni_42−*x*_Co_*x*_Sn_8_ ribbons, the paramagnetic to ferromagnetic transition is successfully introduced when the Co content is higher than 2 at.%, resulting in a stable temperature region for ferromagnetic austenite. The martensitic transformation can be tuned over a wide temperature interval, being accompanied with a large magnetization difference (*i.e.* magnetostructural transformation) when the Co content varies from 3 to 9 at.%. As revealed by the phase diagram of the martensitic transformation and magnetic transition for Mn_50_Ni_42−*x*_Co_*x*_Sn_8_ melt-spun ribbons, the magnetostructural transformation can occur within the temperature range from 355 to 222 K, below the Curie temperature of the austenitic phase 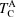, which varies from 363 to 534 K. Thus, a 133 K temperature window for the coupled magnetostructural transformation is established.

**Figure 12:**
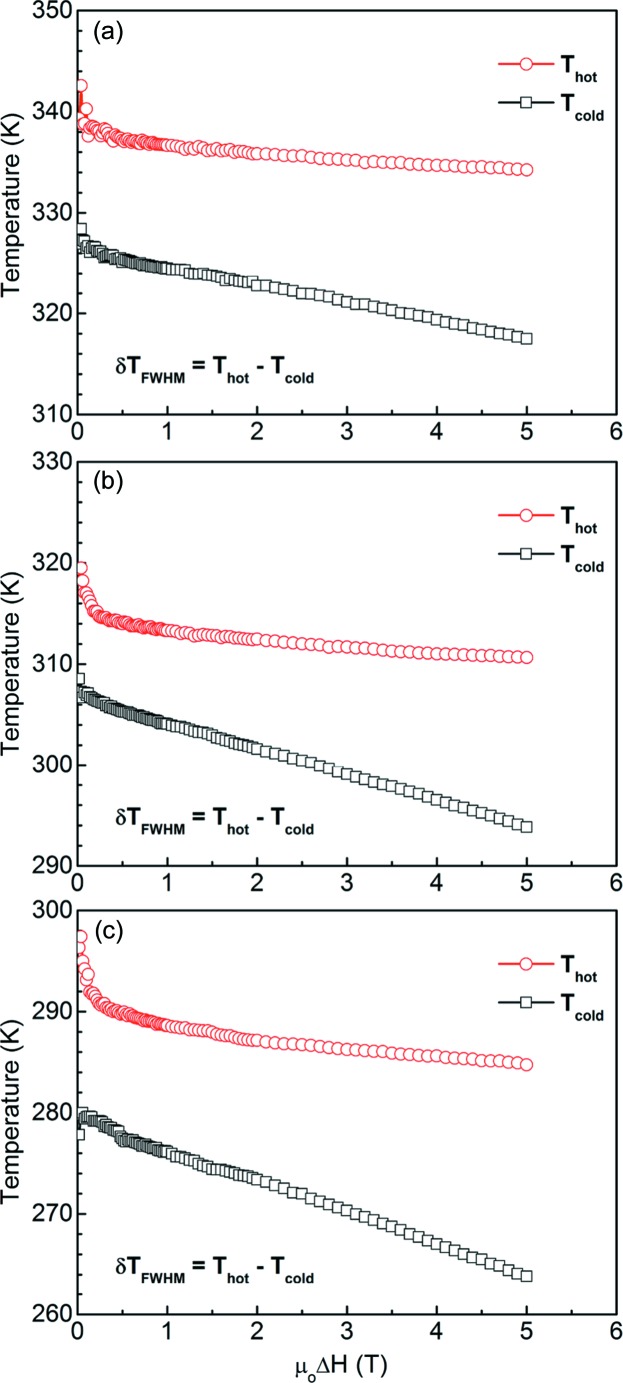
The field dependence of the temperatures *T*_hot_ and *T*_cold_ that define the δ*T*_FWHM_ for (*a*) Mn_50_Ni_36_Co_6_Sn_8_, (*b*) Mn_50_Ni_35_Co_7_Sn_8_ and (*c*) Mn_50_Ni_34_Co_8_Sn_8_ ribbon samples.

As demonstrated before, due to the enhanced ferro­magnetic exchange interactions in austenite through the addition of Co, the Δ*M* accompanying the martensitic transformation is greatly enlarged, resulting in a large 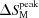 across the magnetostructural transformation. Moreover, the enhanced Δ*M* gives rise to a large magnetic field dependence of the transformation temperatures, which results in the occurrence of a field-induced inverse martensitic transformation from a weakly magnetic martensite to a ferromagnetic austenite. Owing to this effect, the Δ*S*_M_ curves are broadened towards the lower temperature region with increasing magnetic field change. Fig. 12 ▸ displays the field dependence of the temperatures *T*_hot_ and *T*_cold_ that define the working temperature range δ*T*_FWHM_ (δ*T*_FWHM_ = *T*_hot_ − *T*_cold_) of the Δ*S*_M_(*T*) curves for the ribbons with *x* = 6, 7 and 8. The figure shows how δ*T*_FWHM_ is gradually widened with increasing magnetic field change. Consequently, the refrigeration capacity is greatly increased due to the enlarged working temperature range.

For the first-order magnetostructural transformation, transformation hysteresis is unavoidable, resulting in detrimental hysteresis losses. Such irreversible losses directly impair the efficiency of magnetic refrigeration applications. Reduction of hysteresis losses and enhancement of cyclability are of great importance for practical applications. In the present ribbons, it is shown that there are significant hysteresis losses and they increase with increasing Co content. The typical hysteresis behaviour should be closely related to microstructural features as well as the intrinsic lattice distortion. On one hand, the refined grains in the ribbons result in the formation of quite a large number of grain boundaries of initial austenite, which would evidently increase the resistance of the phase transformation and the corresponding transformation hysteresis. Thus, further post-heat treatments aimed at increasing the grain size and reducing the grain boundaries could be helpful to reduce the hysteresis losses in the ribbons. On the other hand, the hysteresis behaviour could also be ascribed to the significant lattice distortion and the corresponding unit-cell volume variation associated with the magnetostructural transformation. The lattice distortion along the *b*_M_ axis [*i.e.* (*b*_M_/*a*_A_) − 1] gradually increases with increasing Co content, since *b*_M_ of martensite tends to decrease whereas *a*_A_ of austenite increases. This should account for the increasing hysteresis losses with increasing Co content. Thus, proper compositional tuning or alloying to lower the lattice distortion and improve the lattice compatibility could be an effective method of reducing hysteresis losses. In addition, the hysteresis behaviour can also be manipulated by applying an additional external field other than a magnetic field, such as hydro­static pressure (Liu *et al.*, 2012 ▸). As demonstrated for an Ni–Mn–In–Co alloy, the hysteresis behaviour can be significantly reduced when the sample is magnetized without bias stress but demagnetized under a low external hydro­static pressure of 1.3 kbar (1 bar = 100 000 Pa; Liu *et al.*, 2012 ▸). Such an external stimulus would provide an additional driving force to assist the field-induced austenite to transform back to martensite.

## 5. Conclusions   

In this work, high Mn-content Mn_50_Ni_42−*x*_Co_*x*_Sn_8_ melt-spun ribbons with 0 ≤ *x* ≤ 10 were prepared and their crystal structures, microstructures, magnetostructural transitions and magnetocaloric properties were systematically investigated. From the results obtained, the following conclusions can be drawn:

(i) Martensite in Mn_50_Ni_42−*x*_Co_*x*_Sn_8_ ribbons possesses the 6*M* type monoclinic crystal structure, whereas the austenite has a cubic *L*2_1_ structure. TEM examinations reveal that the 6*M* martensite plates exhibit stacking faults as a substructure, and there are four types of twin-related martensite variants distributed alternately within one variant colony.

(ii) A decrease in the electron concentration with Co substituted for Ni results in a gradual decrease in the martensitic transformation temperatures. When the Co content is higher than 2 at.%, a paramagnetic to ferromagnetic transition of austenite appears and 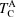 increases with increasing Co content. For ribbons with a Co content from 3 to 9 at.%, the martensitic transformation occurs from ferromagnetic austenite to weakly magnetic martensite. The ribbons exhibit strong magnetostructural coupling over a wide temperature range from 355 to 222 K. Owing to the large magnetization difference, the magnetic field can greatly reduce the martensitic transformation temperatures, resulting in the occurrence of field-induced inverse martensitic transformation.

(iii) Ribbon samples with a Co content from 6 to 8 at.% exhibit a significant magnetic entropy change. Under a magnetic field change of 5 T, the 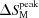 values for the Mn_50_Ni_36_Co_6_Sn_8_, Mn_50_Ni_35_Co_7_Sn_8_ and Mn_50_Ni_34_Co_8_Sn_8_ ribbons are 14.1, 18.6 and 16.0 J kg^−1^ K^−1^, respectively, and the effective refrigerant capacities *RC*_eff_ are 143, 175 and 178 J kg^−1^, respectively. The values of Δ*S*_M_ and *RC*_eff_ are comparable with or even superior to those of Ni-rich Ni–Mn-based polycrystalline bulk alloys.

Compared with the ternary Mn–Ni–Sn alloys, the addition of Co can realise strong magnetostructural coupling over a wide temperature range with enhanced magnetocaloric properties in Mn_50_Ni_42−*x*_Co_*x*_Sn_8_ melt-spun ribbons. Therefore, they can be considered as promising candidates for magnetic refrigeration.

## Figures and Tables

**Table 1:** EDS results for Mn_50_Ni_42−*x*_Co_*x*_Sn_8_ (*x* = 0–10) melt-spun ribbons

	Actual composition (at.%)			
Nominal composition	Mn	Ni	Co	Sn
Mn_50_Ni_42_Sn_8_	50.4	41.4	0	8.2
Mn_50_Ni_41_Co_1_Sn_8_	50.1	40.7	1.1	8.1
Mn_50_Ni_40_Co_2_Sn_8_	50.3	39.4	2.1	8.2
Mn_50_Ni_39_Co_3_Sn_8_	49.8	38.6	3.6	8.0
Mn_50_Ni_38_Co_4_Sn_8_	50.5	37.2	4.1	8.2
Mn_50_Ni_37_Co_5_Sn_8_	50.6	36.2	4.9	8.3
Mn_50_Ni_36_Co_6_Sn_8_	49.9	35.6	6.2	8.3
Mn_50_Ni_35_Co_7_Sn_8_	50.3	34.4	6.8	8.5
Mn_50_Ni_34_Co_8_Sn_8_	50.7	33.6	7.7	8.1
Mn_50_Ni_33_Co_9_Sn_8_	50.1	32.8	9.0	8.1
Mn_50_Ni_32_Co_10_Sn_8_	50.7	31.5	9.8	8.1

**Table 2:** Maximum magnetic entropy change (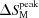), refrigerant capacity (*RC*), average hysteresis loss (〈*HL*〉) and effective refrigerant capacity (*RC*_eff_) values for Mn_50_Ni_42−*x*_Co_*x*_Sn_8_ (*x* = 6–8) ribbons under field changes of 2 and 5 T

	Mn_50_Ni_36_Co_6_Sn_8_		Mn_50_Ni_35_Co_7_Sn_8_		Mn_50_Ni_34_Co_8_Sn_8_	
	2 T	5 T	2 T	5 T	2 T	5 T
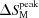 (J kg^−1^ K^−1^)	6.4	14.0	10.0	18.6	8.5	16.1
*RC* (J kg^−1^)	65	189	86	259	90	273
〈*HL*〉 (J kg^−1^)	8	46	17	84	23	95
*RC*_eff_ (J kg^−1^)	57	143	69	175	67	178
